# Identification, Distribution and Population Dynamics of *Francisella*-like Endosymbiont in *Haemaphysalis doenitzi* (Acari: Ixodidae)

**DOI:** 10.1038/srep35178

**Published:** 2016-10-12

**Authors:** Jian-Nan Liu, Zhi-Jun Yu, Li-Meng Liu, Ning-Xin Li, Rong-Rong Wang, Chun-Mian Zhang, Jing-Ze Liu

**Affiliations:** 1 Key Laboratory of Animal Physiology, Biochemistry and Molecular Biology of Hebei Province, College of Life Sciences, Hebei Normal University, No. 20 Nanerhuan Eastern Road, Shijiazhuang, Hebei, 050024, P. R. China; 2 Department of Integrative Biology, University of California, Berkeley, Valley Life Sciences Building, Room 5155A, Berkeley, CA, 94720-3140, USA

## Abstract

*Francisella-*like endosymbionts (FLEs) with significant homology to *Francisella tularensis* (γ-proteobacteria) have been characterized in several tick species, whereas knowledge on their distribution and population dynamics in ticks remains meager. Hence, in the current study, we identified a novel *Francisella*-like endosymbiont (FLEs-Hd) from the tick *Haemaphysalis doenitzi* and evaluated the putative functions of this symbiont. Results indicated that FLEs-Hd had 100% infection rate and a perfect vertical transmission in *H. doenitzi*, and that it is distributed in ovaries, malpighian tubules, salivary glands and midguts of the ticks, suggesting that FLEs-Hd presumably is a crucial symbiont of the host without specific tissue tropism. To further explore the function of the symbiont, the population dynamics of FLEs-Hd at each developmental stage of ticks and in tissues at different reproductive statuses were determined by real-time quantitative polymerase chain reaction (real-time qPCR). Results showed that the high density and regular population dynamics of FLEs-Hd appeared in female ovaries, suggesting that the symbiont may provide necessary nutrients or regulators to ensure normal ovary development of ticks.

*Francisella* is an expanding genus of the gamma subdivision of the Proteobacteria with a facultative intracellular life style^1^. Four valid species, including *Francisella tularensis, F. philomiragia, F. noatunensis*, and *F. hispaniensis,* have been recognized within this genus^2^,^3^. Among them, *F. tularensis* is ascribed as category A etiologic agent of bioterrorism, which can cause the highly infectious zoonotic disease tularaemia^4^,^5^ through multiple infection routes^6^,^7^,^8^. The tick *Dermacentor andersoni* was first implicated as a vector of pathogenic *F. tularensis*^9^, and then *Francisella*-like endosymbionts (FLEs) were also described in this tick species^10^. Up to now, many FLEs closely related to *F. tularensis* have been identified in ticks belonging to *Ornithodoros*^11^,^12^, *Dermacentor*^10^,^13^, *Amblyomma*^14^, *Anocentor*^15^, *Hyalomma*^16^, *Rhipicephalus*^16^ and *Ixodes* (Genbank accession No. JQ740890), whereas no FLEs have yet been identified from ticks within the genus *Haemaphysalis*.

Symbionts of ticks are capable of providing some fitness advantages for host ticks which therefore impact the population and community dynamics of ticks by altering their ecology. For instance, *Coxiella*-like symbiont might impact the reproduction of *Amblyomma americanum*^17^,^18^,^19^; *Candidatus* Midichloria mitochondrii, the symbionts of *Ixodes ricinus*, is coupled with the process of engorgement and molt^20^,^21^, and *Rickettsia peacockii* colonizing *D. variabilis* and *D. andersoni* could defend against pathogens invasion^22^,^23^. As one of the common tick symbionts, FLEs are intracellular and can be transmitted transovarially^16^, and have been mainly found in reproductive tissues of female ticks^14^. But a FLE of *D. variabilis* was also potentially colonized in salivary glands^24^. However, the population dynamics and functions of FLEs have not been evaluated yet.

In the present study, the compositions of bacterial communities in *Haemaphysalis doenitzi* were analyzed with the bacterial 16S rRNA gene library and restriction fragment length polymorphism (RFLP). Symbionts were characterized using diagnostic polymerase chain reaction (diagnostic PCR) and transmission electron microscope (TEM). Additionally, the functions of the putative symbiont were also evaluated preliminarily by assaying the population dynamics of the symbiont using real-time quantitative PCR (real-time qPCR), which will provide basic knowledge for further clarification of the relationship between FLEs and the host ticks.

## Results

### Diversity of bacterial communities in *H. doenitzi*

Three different bacterial genera were detected from *H. doenitzi* by bacterial 16S rRNA gene library and RFLP analysis, including *Sphingomonas* (alpha-Proteobacteria) with 99.5% similarity to *Sphingomonas melonis* (Genbank accession No. NR_028626)^25^, *Acinetobacter* showing 99.3% similarity to *Acinetobacter beijerinckii* (Genbank accession No. NZ_BBTL01000030) and *Francisella*-like symbiotic bacteria. The *Francisella* 16S rRNA sequences amplified from *H. doenitzi* exhibited 99% identity (10 different nucleotides) with the FLEs of *Ornithodoros moubata* (Genbank accession No. AB001522) (Supplementary Fig. 1) and were designated as FLEs-Hd.

### Phylogenetic analysis

**Figure 1:**
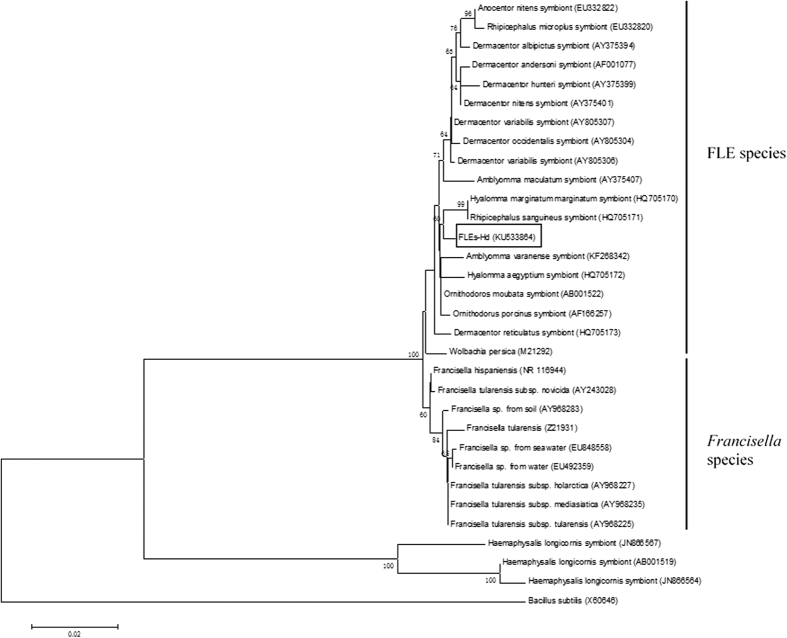
Neighbor-Joining unrooted tree showing genetic similarity between the 16S rDNA gene of *Francisella*-like endosymbiont isolated from *H. doenitzi* and GenBank records. The percentage of replicate trees in which the associated taxa clustered together in the bootstrap test (1000 replicates) is shown next to the branches. Genetic distance was computed using the Kimura 2-parameter method and are in the units of the number of base substitutions per site. Sequence alignments and tree generation were conducted in MEGA6.

The partial 16S rRNA gene sequence-based phylogenetic reconstruction showed that FLEs-Hd was relatively closely related to other symbiotic *Francisella*, whereas separated from the pathogenic *Francisella* species (Fig. 1). Furthermore, the FLEs-Hd clustered with the FLEs of *Hyalomma marginatum marginatum* and *Rhipicephalus sanguineus*, but separately from the clade comprising isolates of *H. longicornis* symbionts.

### Prevalence and Transmission

**Figure 2:**
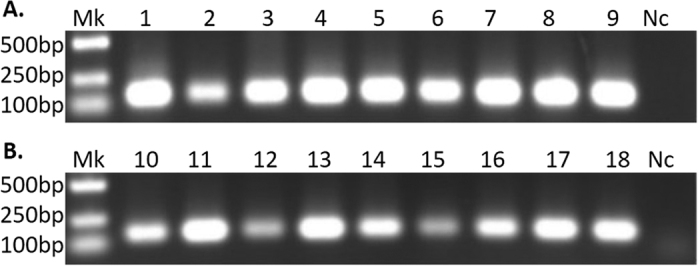
The Infection rate detection of FLEs-Hd in field colony of *H. doenitzi*. Lanes 1 to 11 (**A**) Mk, DNA Marker; 1, female No. 1; 2, female No. 2; 3, female No. 3; 4; female No. 4; 5, female No. 5; 6, female No. 6; 7, female No. 7; 8, female No. 8; F9, female No. 9; Nc, negative control. Lanes 1 to 11 (**B**) Mk, DNA Marker; 10, male No. 1; 11, male No. 2; 12, male No. 3; 13; male No. 4; 14, male No. 5; 15, male No. 6; 16, male No. 7; 17, male No. 8; 18, male No. 9; Nc, negative control. The gels were run under the same experimental conditions. Cropped gels are presented (Full-length gels are shown in Supplementary Fig. 2).

**Figure 3:**
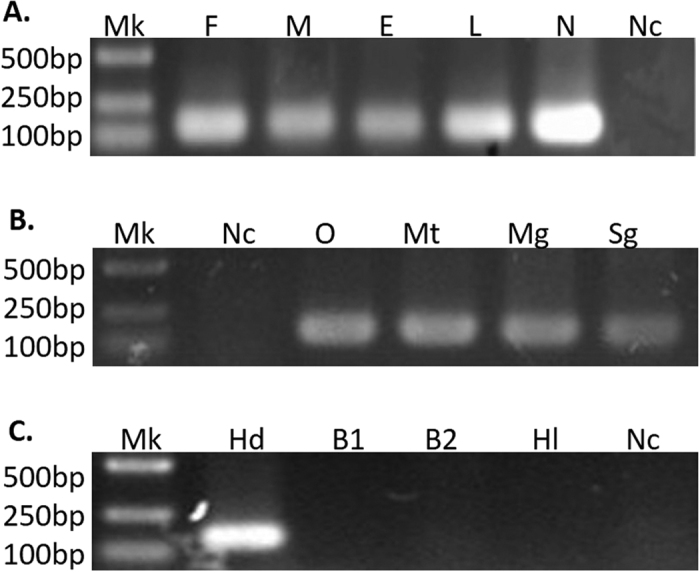
The vertical transmission detection of FLEs-Hd at different developmental stages of *H. doenitzi*. Lanes 1 to 7 (**A**) Mk, DNA Marker; F, females; M, males; E, eggs; L, larvae; N, nymphs; Nc, negative control. Detection of infection sites of FLEs-Hd in different tissues of *H. doenitzi*. Lanes 1 to 6 (**B**) Mk, DNA Marker; Nc, negative control; O, ovaries; Mt, malpighian tubules; Mg, midguts; Sg, salivary glands. The horizontal transmission detection of FLEs-Hd in host animal and *H. longicornis* collected from the same region as *H. doenitzi*. Lanes 1 to 6 (**C**) Mk, DNA Marker; Hd, *H. doenitzi*; B1, blood of host animal before tick ingested; B2, blood of host animal after tick ingested; Hl, *H. longicornis*; Nc, negative control. The gels were run under the same experimental conditions. Cropped gels are presented (Full-length gels are shown in Supplementary Fig. 3).

A total of 20 females and 20 males from field colony were collected and studied by diagnostic PCR and sequencing. The results showed that all the ticks were FLEs-Hd positive, suggesting that the infection rate of FLEs-Hd is 100% in *H. doenitzi* (Fig. 2, Supplementary Fig. 2). To test whether transmission of FLEs-Hd was transovarial or transstadial, samples of eggs, the first generation (F1) larvae, F1 nymphs, F1 females and F1 males were screened. All the tick extractives were infected with FLEs-Hd, which is consistent with vertical transmission (Fig. 3A, Supplementary Fig. 3A). For assessing the efficiency of vertical transmission, the sixth generation (F6) ticks from laboratory colony were examined by PCR. All the samples were FLEs-Hd positive, indicating that the vertical transmission efficiency of FLEs-Hd in laboratory colony was 100%. The distribution analysis revealed that FLEs-Hd was harbored in ovaries, malpighian tubules, salivary glands and midguts, but no specific tissue tropism was observed (Fig. 3B, Supplementary Fig. 3B). No FLEs-Hd was detected from blood samples of the tick hosts (*Oryctolagus cuniculus*), regardless of whether the host animals were attacked by *H. doenitzi* carrying FLEs-Hd. Additionally, no FLEs were detected in the field colony of *H. longicornis*, which has an overlapping occurrence with *H. doenitzi* (Fig. 3C, Supplementary Fig. 3C).

### Population dynamics of FLEs-Hd in tick development

**Figure 4:**
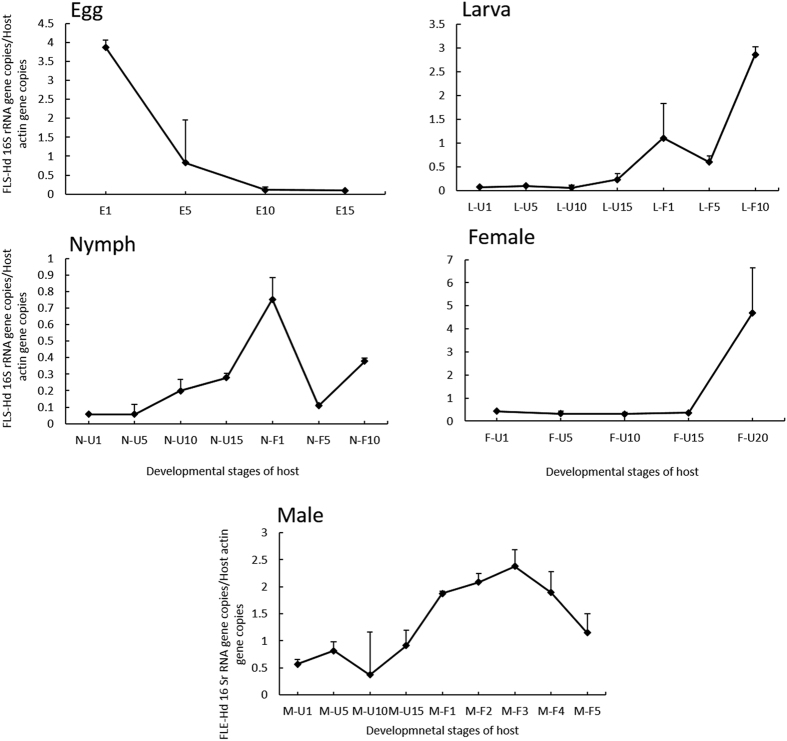
Population dynamics of FLEs-Hd in various *H. doenitzi* developmental stages. Abbreviations: E1: eggs day 1; E5: eggs day 5; E10: eggs day 10; E15: eggs day 15; L-U1: unfed larvae day 1: L-U5: unfed larvae day 5; L-U10: unfed larvae day 10; L-U15: unfed larvae day 15; L-F1: fed larvae day 1; L-F5: fed larvae day 5; L-F10: fed larvae day 10; N-U1: unfed nymphs day 1: N-U5: unfed nymphs day 5; N-U10: unfed nymphs day 10; N-U15: unfed nymphs day 15; N-F1: fed nymphs day 1; N-F5: fed nymphs day 5 N-F10: fed nymphs day 10; F-U1: unfed females day 1: F-U5: unfed females day 5; F-U10: unfed females day 10; F-U15: unfed females day 15; F-U20: unfed females day 20; M-U1: unfed male day 1: M-U5: unfed male day 5; M-U10: unfed male day 10; M-U15: unfed male day 15; M-F1: fed male day 1; M-F2: fed male day 2; M-F3: fed male day 3; M-F4: fed male day 4; M-F5: fed male day 5. Means and standard errors of means are shown.

The density of FLEs-Hd was highest in the first-day laid eggs, then decreased significantly, and kept the low level till eclosion (Fig. 4 Egg). In larvae and nymphs, the population dynamic of FLEs-Hd exhibited a similar tendency. The densities were at a lower level in non-engorged stage, and increased on the first day after engorgement, subsequently declined slightly on the 5^th^ day after engorgement, and then increased again (Fig. 4 Larva, Nymph). In female, the prominent peak appeared at the 20^th^ day of non-engorged stage (Fig. 4 Female). The density of FLEs-Hd in males was at lower level in non-engorged stage and increased after engorgement (Fig. 4 Male). As a whole, the density of FLEs-Hd was lower in first day laid eggs than in unfed females on the 20^th^ day of non-engorged stage, but was higher than in larvae, nymphs and males.

### Population dynamics of FLEs-Hd in tissues of ticks at different feeding statuses

**Figure 5:**
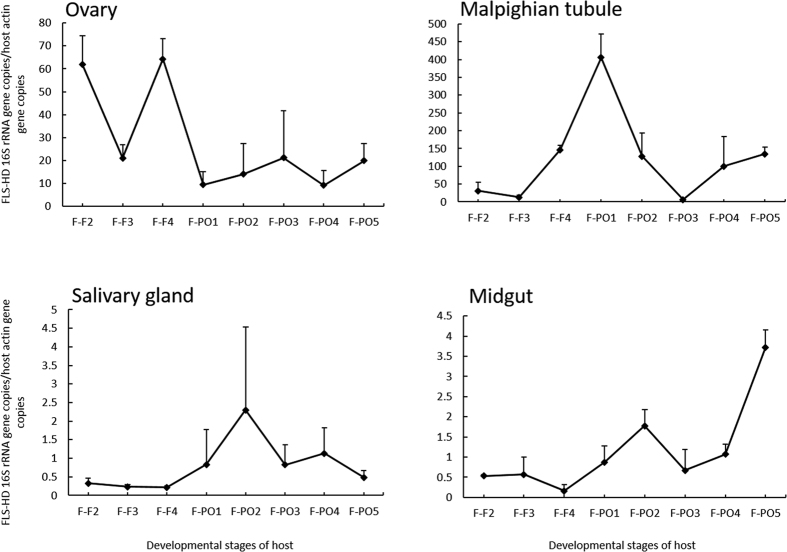
Population dynamics of FLEs-Hd in different tissues (ovaries, malpighian tubules, midguts, and salivary glands) of mated females in feeding and preovipositon stages. Abbreviations: F-F2: feeding stage day 2; F-F3: feeding stage day 3; F-F4: feeding stage day 4; F-PO1: preovipostion stage day 1; F-PO2: preovipostion stage day 2; F-PO3: preovipostion stage day 3; F-PO4: preovipostion stage day 4; F-PO5: preovipostion stage day 5. Means and standard errors of means are shown.

In ovaries, the density of FLEs-Hd reached a higher value on the second day of feeding when ticks started to suck blood from the host, and declined abruptly from the second day to the third day. A second boost in the density was on the fourth day when ticks were approaching engorgement, declined again and kept a low level in the remaining stages (Fig. 5 Ovary). In contrast, the peak value of the symbiont density in malpighian tubules appeared on the first day in preovipositon stage, then declined gradually and reached a relatively lower level on the third day in preovipositon stage (Fig. 5 Malpighian tubule). In midgut and salivary glands, the densities of FLEs-Hd were lower than those in ovary and malpighian tubule, although they showed a small increase on the second day at preovipositon stage (Fig. 5 Salivary gland, Midgut).

### Visualization of symbiont structures by TEM

**Figure 6:**
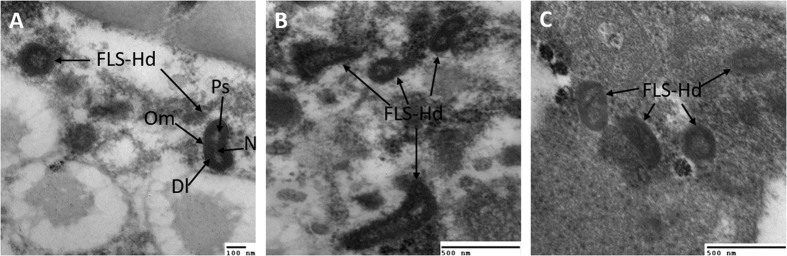
Transmission electron micrographs of *H. doenitzi* tissue cells infected with the FLEs-Hd. (**A,B**) Pleomorphic FLEs-Hd infest in host ovaries and reproduce through binary fission. (**C**) FLEs-Hd inhabited in the malpighian tubules of tick. Abbreviation indicates: Om, outer membrane; Dl, Dense layer; Ps, periplasmic space; N, nucleoplasm.

Under TEM, FLEs-Hd showed three membranes when samples were examined at higher magnification (Fig. 6A), whereas bacterial cells in ovaries showed polymorphous shapes (Fig. 6B). In malpighian tubules, the morphology of FLEs-Hd was strikingly similar in shape and size to that in ovaries (Fig. 6C).

## Discussion

The microbial community of the tick *H. doenitzi* was explored using 16S rRNA in the current study. *Sphingomonas* and *Acinetobacter* were characterized as the main bacteria in *H. doenitzi*, and a novel *Francisella*-like endosymbiont was identified and further analyzed*. Sphingomonas* bacteria have been reported in *I. ricinus, I. ovatus, I. persulcatus* and *H. flava* by assays of bacterial communities^26^,^27^. *Acinetobacter* is known as environmental contaminant commonly found in soil, and some *Acinetobacter* species that are typical representatives of nosocomial infections have been found in ticks^28^. Although *Acinetobacter* has been regarded as the suspected symbiont in some arthropods^29^,^30^,^31^, the *Acinetobacter* identified in the current study did not exhibit mutualistic relationship with host, since no positive result of *Acinetobacter* was detected in the laboratory colony of *H. doenitzi*.

The nucleotide sequence of 16S rRNA gene of the novel FLEs-Hd was 99% similarity to the FLEs of *O. moubata* with a difference of 10 nucleotides. Furthermore, it formed an obvious individual clade in the phylogenetic tree, though it was grouped together with the FLEs of *Hy. m. marginatum and R. sanguineus*. Phylogenetic analysis of partial 16S rRNA gene sequences of *Francisella* spp. highlighted that FLEs-Hd is more closely related to the other symbiotic *Francisella* than to the virulent *Francisella* spp., suggesting that FLEs-Hd is likely nonpathogenic. The deficiency of RD1 sequence in FLEs might be as a marker of differentiation *F. tularensis* from FLEs^16^,^32^, but the pathogenic potential of FLEs remains mostly undefined. Simultaneously, a relatively distant evolutionary relationship was observed between FLEs-Hd and the symbionts of *H. longicornis,* which is genetically close to *H. doenitzi*, and this was consistent with the previous hypothesis that FLEs may not co-evolve with their respective host ticks^14^,^15^,^33^,^34^.

In this study, the 100% prevalence rate of FLEs-Hd in field colony is significantly higher than those reported previously in other tick species^14^,^16^. There is also a fantastically stable vertical transmission of FLEs-Hd throughout the entire life cycle of *H. doenitzi*, which is in accordance with the FLEs in *D. variabilis* and *D. albipictus*^26^,^35^. These data demonstrate that FLEs is an important symbiont and may contribute to the fitness of *H. doenitzi*. Interestingly, no FLEs were detected in the field colony of *H. longicornis*, which has an overlapping occurrence with *H. doenitzi*. Moreover, such endosymbionts were not detected in blood from host animals after infestation with *H. doenitzi* carrying FLEs-Hd, suggesting that FLEs may not be transmitted to the host animal under laboratory conditions and may not have any pathogenicity.

In *H. doenitzi,* the density of FLEs-Hd is not invariable, but rather changing regularly with the development of host. The significant drop in the density of FLEs-Hd from egg to larval stage is probably due to the bottleneck effect during vertical transmission. Similar phenomenon was also observed in the intracellular symbiont of *Ca.* Midichloria mitochondrii in *I. ricinus*^20^,^21^. The narrow bottleneck could give rise to more genetic drift in symbiont populations, further leads to genome erosion or streamlining, thereafter affect the process of life activities of hosts^36^,^37^,^38^,^39^,^40^. In fact, numerous insect symbionts population also experience the similar phenomenon, for example, *Buchnera* was reduced when it was transmitted from *Acyrthosiphon pisum* embryo to the first instar nymph, and the density decrease is expected to have important consequences for the evolutionary genetics of the symbiont^41^. In stinkbug, only 1/10 of *Ca.* Ishikawaella capsulate transmitted to a newborn nymph from mother, which can be expected to further augment the deleterious effects^42^, and *Ca.* Streptomyces philanthi which inhabits in beewolf show a few number in the cocoon by the emerging female, which may have significant effect on the evolution of the beewolf-Streptomyces symbiosis by increased genetic drift^43^. Furthermore, the density of FLEs-Hd strikingly increased following the blood-feeding of larvae, nymphs and males, similar to the dynamics of *Ca.* Midichloria mitochondrii in *I. ricinus*^20^, suggesting that the symbionts are presumably beneficial to blood-sucking of ticks.

In ovaries, the density of the symbionts was higher at the initial feeding stage (2^nd^ day of feeding) and at almost engorged female (4^th^ day of feeding) and then dropped to a stable lower level at next stages. In fact, the steps of multiplication coincide with developmental phases of ovary. The primary oocytes appear and grow gradually after female emerged; oocytes start to develop immediately when females begin to suck blood; vitellogenesis is initiated after mating^44^. The multiplication of symbionts at definite developmental stages of the host may be indicative of those phases in which the symbiosis is of the greatest importance^43^. Therefore, it is speculated that the FLEs-Hd may be closely related to the initial development of ovary of the host and may provide some necessary nutrients or regulators to ensure the ovary development. Simultaneously, besides digesting blood meal, midguts are also the location of vitellogenin synthesis in many tick species^45^,^46^,^47^. Coincidentally, the growth of symbionts in midgut was observed at both the beginning of vitellogenin synthesis and the last day before oviposition, further suggesting that the FLEs-Hd may be related to the reproduction of the host. In striking contrast, the density of symbionts in malpighian tubules is upon reaching the maximum level on the first day of engorgement of females. The changes may be caused by the transference of most symbionts from ovary to malpighian tubules, and the elimination of symbionts from tick with excrements.

In this work, the FLEs-Hd in ovaries and malpighian tubules of *H. doenitzi* showed no vesicles or other vacuolar membranes, and the morphology is in accordance with the symbiont *F. tularensis* in *I. ricinus*^48^, suggesting a more direct interaction and exchange between the host and FLEs. Taken together, our study identified a novel FLEs in *H. doenitzi*, and the high prevalence and stable vertical transmission of this symbiont suggested it would be the primary symbiont in *H. doenitzi*. The bottleneck of FLEs-Hd was present in vertical transmission, whereas no tissue tropism and horizontal transmission of FLEs-Hd were detected. From the regular population dynamics of FLEs-Hd in the ovary of *H. doenitzi*, this symbiont was tentatively associated with the ovary development and reproduction of ticks. However, further investigations are required to explore the mechanisms underlying the interaction between FLEs and ticks, which will contribute to the subsequent biocontrol of ticks.

## Methods

### Ethics Statement

The protocol of all animal experiments was approved by the Institutional Animal Care and Use Committee of Hebei Normal University. And all methods were carried out in accordance with the relevant approved guidelines and regulations.

### Collection and rearing of ticks

*H. doenitzi* ticks were originally collected by blanket dragging in Cangxi (31°37′–32°10′N, 105°43′–106°28′E) of Sichuan province. Part of collected ticks (defined as field colony) were placed in sealed vials and preserved at −80 °C until use; others were reared on domestic rabbits *Oryctolagus cuniculus* as previously described^49^. Offspring of *H. doenitzi* (defined as laboratory colony) were maintained at 26 °C, humidity 80% with a light: dark regime of 16:8 hr.

### Tick dissection and DNA extraction

Total genomic DNA for constructing bacterial 16S rRNA gene libraries were extracted from each group of adults (10 females and 10 males, respectively). Additionally, to investigate the prevalence, vertical transmission and the population dynamics of symbiont using real-time qPCR, DNA was isolated from individual adults in field colony and pooled ticks from laboratory colony at other developmental stages (500 eggs, 200 larvae, and 50 nymphs, respectively).

To assess infection sites of symbiont, tissues, including ovaries, malpighian tubules, salivary glands and midguts, were dissected from females at different feeding statuses. Tissue dissection was performed sterilely under a stereomicroscope at 10 × 23 magnification using a micro-clipper in sterile phosphate-buffered saline (PBS)(137 mM NaCl, 2.7 mM KCl, 4.3 mM Na_2_HPO_4_·7H_2_O, 1.4 mM KH_2_PO_4_, pH 7.4) as described previously^50^. Tissues were collected in 1.5 ml sterile vials (Axygen, USA) respectively, and then genomic DNA was extracted as described in the DNeasy Blood & Tissue Kit manual (Qiagen, Germany). Extracted genomic DNA was stored at −20 °C until analysis. The DNA concentration was measured using a NanoDrop 1000 spectrophotometer (Thermo Scientific, USA).

### Construction of bacterial 16S rRNA gene library and RFLP analysis

The bacterial 16S rRNA gene library was constructed by amplifying about 1500 bp fragment of 16S rRNA gene using bacterial universal primers Eub27F/Eub1492R^51^. Primer sequences used for each PCR reaction are described in Table 1. PCR products were purified by PCR Purification Kit (Bioteke, China) and ligated into pEASY-T1 cloning vector using pEASY-T1 simple cloning kit (TransGen, China) according to the manufacturer’s protocol. DNA recombinant was transformed into *Escherichia coli* TOP10 competent Cell (TransGen, China). Subsequently, the bacterial 16S rRNA gene library was digested by both *Hae* III and *Rsa* I restriction endonucleases for RFLP analysis. Positive clones with different restriction fragment patterns were sequenced by sequencing company (Sangon Biotech, China).

### Identification of symbiont

Diagnostic PCR assays were performed with two sets of specific primers (Table 1). PCR reactions were carried out in 20 μl volume containing 20 mM Tris-HCl (pH 8.4), 50 mM KCl, 1.5 mM MgCl_2_, 200 μM of each dNTP, 2.5 U Platinum *Taq* DNA polymerase (Invitrogen, USA), 0.5 mM each primer and 2 μl of template DNA. PCR reaction was performed as: 1 cycle of 94 °C for 2 min, followed by 30 cycles of 94 °C for 30 s, 56 °C for 30 s, and 72 °C for 15 s, with a final extension at 72 °C for 10 min. Nuclease free water was used as negative control during each set of PCR reactions. PCR products were electrophoresed in 1% agarose gels and visualized using AlphaImager HP (Alpha Innotech, USA), and the fragments with appropriate size were cut from the gel and purified with DNA gel extraction kit (Bioteke, China). To confirm the results, the purified amplicons were randomly selected, cloned, sequenced, and checked by BLAST search.

### Phylogenetic analysis

Sequence of FLEs-Hd was compared with known sequences listed in the GenBank nucleotide sequence databases. The gram-positive bacterium *Bacillus subtilis* (X60646) was used as an outgroup species. The sequences obtained were aligned using the CLUSTAL W program and manually checked with excluded DNA gaps^52^. The phylogenetic trees were produced according to the neighbor-joining method after Kimura 2-parameter correction in the MEGA version 6 using bootstrap analyses with 1000 replicates^53^.

### Quantitative Real-time PCR

SYBR green-based real-time qPCR specific for FLEs-Hd was used to monitor the population dynamics of symbiont loads in various tissues and developmental stages of ticks. Standard curves were created with serial dilutions of plasmids containing inserts of the amplified specific 16S rRNA gene sequences from symbionts and actin gene sequences (Supplementary Fig. 4). The amplification efficiencies of primers FLEs F/R and Actin F/R were 90.1% and 100%, respectively. A master mix was prepared using 12.5 μl of 2X TransStart^TM^ Top Green qPCR SuperMix (TransGen, China), 0.5 μl of each 10 μM primer (Table 1), 10.5 μl H_2_O and 1 μl template DNA for a final reaction volume of 25 μl. Briefly, qPCR assays were conducted in 96-well polypropylene plates in a Mx3005P qPCR system (Agilent Technologies, USA). The thermal profile was set as follows: 94 °C for 30 s; 40 cycles of 94 °C for 5 s and 60 °C for 30 s. The primers with high amplification specificity were verified by unique peaks observed in corresponding melting curves. Each plate contained triplicate reactions for each DNA sample. Melting curves were also traced after each assay to confirm that the fluorescence signal had been retrieved from specific PCR products and to ensure the absence of primer dimmers. Sterile water was used as the negative control.

### TEM studies

Dissected ovaries and malpighian tubules from female ticks of laboratory colony infected with FLEs-Hd were studied by TEM examination. Samples were fixed in pre-cooled 0.1 M sodium cacodylate buffer (pH 7.4) containing 2.5% glutaraldehyde and 4% paraformaldehyde at 4 °C overnight. The fixed samples were then washed with PBS buffer and post-fixed in 1% OsO_4_ in the same buffer for 1.5 h at 4 °C. Consequently, all the samples were dehydrated in a graded acetone dehydration series and embedded in Spurr’s resin at 37 °C. Afterwards, samples were polymerized at 65 °C for 48 h. The semi-thin sections (1 μm) for light microscopy were stained with 0.5% toluidine blue; thin sections (80 nm) were mounted on 100-mesh formvar-coated copper grids; samples were stained with 1% uranyl acetate, lead citrate, and bismuth and visualized under a transmission electron microscope (Hitachi HU-11E, Japan) at 75 KV. TEM micrographs were processed using Gatan Digital Micrograph software.

### Nucleotide sequence accession number

The sequence of FLEs-Hd was deposited in GenBank (accession number: KU533864).

## Additional Information

**How to cite this article**: Liu, J.-N. *et al*. Identification, Distribution and Population Dynamics of *Francisella*-like Endosymbiont in *Haemaphysalis doenitzi* (Acari: Ixodidae). *Sci. Rep.* **6**, 35178; doi: 10.1038/srep35178 (2016).

## Supplementary Material

Supplementary Information

## Figures and Tables

**Table 1:** Oligonucleotide primers used for PCR amplification and sequencing.

Primer name	Genera or Species	Target gene	Nucleotide sequence(5′–3′)	Annealing temperature (°C)	Approx products size (bp)	Reference
Eub 27F	Bacteria	16S rRNA	AGAGTTTGATCCTGGCTCAG	55	1500	51
Eub 1492R			TACCTTGTTACGACTT			
153F	*Francisella*	16S rRNA	GCCCATTTGAGGGGGATACC	56	1200	24
1281R			GGACTAAGAGTACCTTTTTGAGT			
Fran 16SqPCR F	*Francisella*	16S rRNA	CAGGACTAGCTTATAGTTGCTG	56	150	This study
Fran 16SqPCR R			CATCTGCGACAGCCTAAAAGC			
Actin qPCRF	*Haemaphysalis*	actin	CCAGACGGAGTACTTGCGC	55	100	50
Actin qPCRR	*Haemaphysalis*	actin	CATGTACCCGGGCATCGCCGA			
