# Supplementary material for: Identification, Distribution and Population Dynamics of Francisella-like Endosymbiont in Haemaphysalis doenitzi (Acari: Ixodidae)
Source: Sci Rep. 2016 Oct 12;6:35178. doi: 10.1038/srep35178 (PMC5059625; doi:10.1038/srep35178)
Supplement: Supplementary Information [file srep35178-s1.pdf]

Identification, Distribution, and Population Dynamics of *Francisella*-like Endosymbiont in *Haemaphysalis doenitzi* (Acari: Ixodidae)

Jian-Nan Liu[1+], Zhi-Jun Yu[1+], Li-Meng Liu[2], Ning-Xin Li[1], Rong-Rong Wang[1], Chun-Mian Zhang[1] & Jing-Ze Liu[1*]

[1]Key Laboratory of Animal Physiology, Biochemistry and Molecular Biology of Hebei Province, College of Life Sciences, Hebei Normal University, No. 20 Nanerhuan Eastern Road, Shijiazhuang, Hebei, 050024, P. R. China

[2] Department of Integrative Biology, University of California, Berkeley, Valley Life Sciences Building, Room 5155A, Berkeley, CA, 94720-3140, United States

*Correspondence and requests for materials should be addressed to J.-Z.L. (email: liujingze@hebtu.edu.cn)

[+]These authors contributed equally to this work.

A

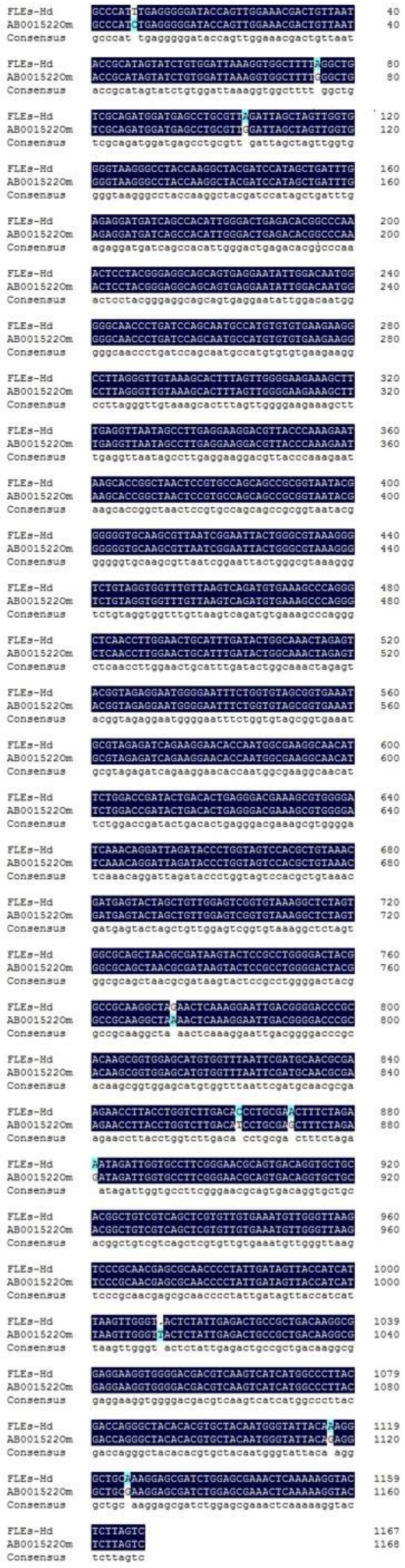

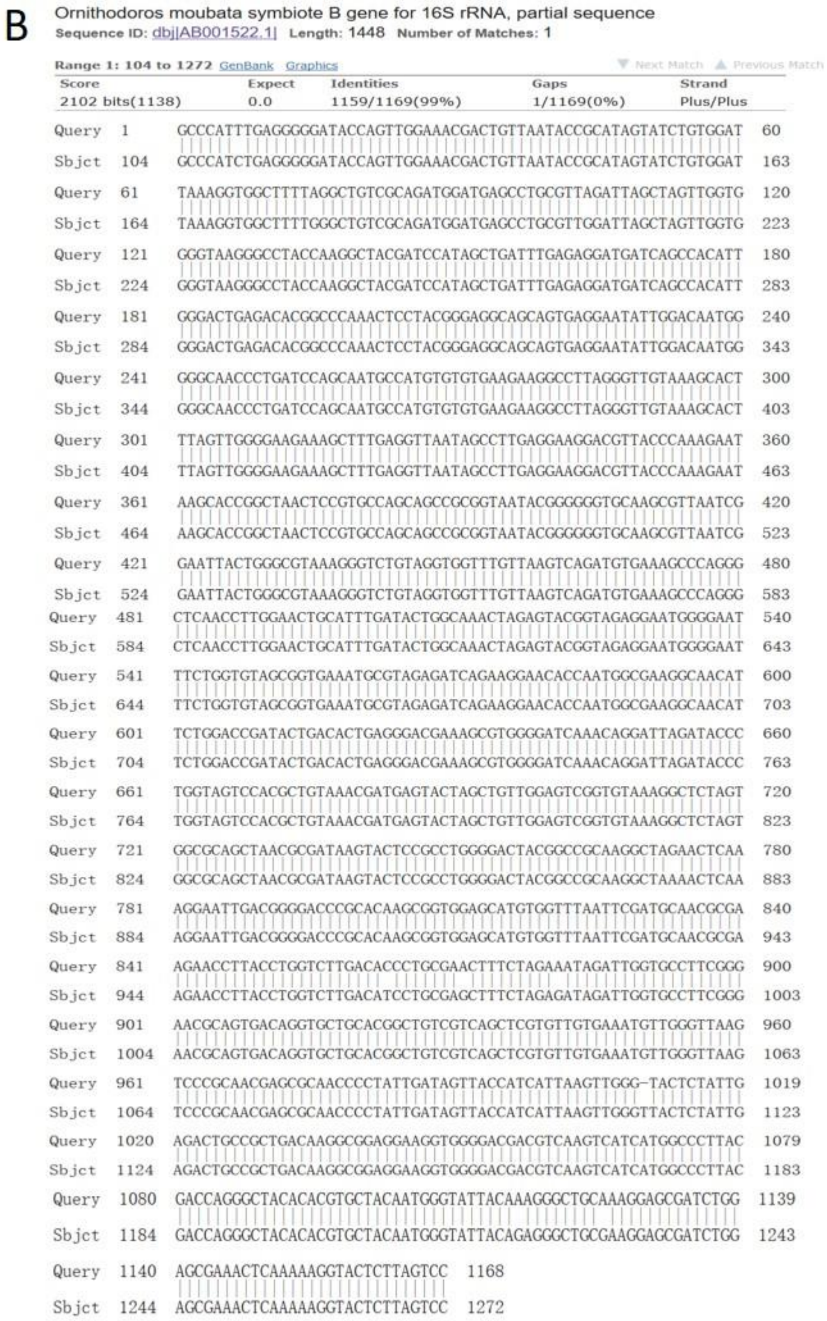

Supplementary Figure 1. Sequence alignment of FLEs-Hd from *H. doenitzi* and FLE form *O. moubata* by DNAMAN8 (A) and BLAST (B).

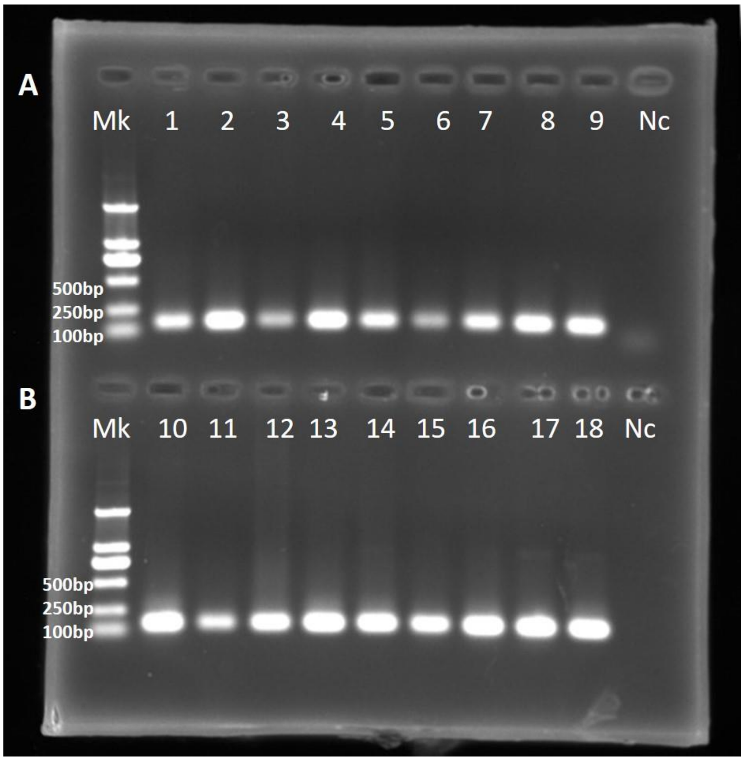

Supplementary Figure 2. The full-length gel of infection rate detection of FLEs-Hd in field colony of *H. doenitzi*. Lanes 1 to 11 (A): Mk, DNA Marker; 1, female No. 1; 2, female No. 2; 3, female No. 3; 4; female No. 4; 5, female No. 5; 6, female No. 6; 7, female No. 7; 8, female No. 8; 9, female No. 9; Nc, negative control. Lanes 1 to 11 (B): Mk, DNA Marker; 10, male No. 1; 11, male No. 2; 12, male No. 3; 13; male No. 4; 14, male No. 5; 15, male No. 6; 16, male No. 7; 17, male No. 8; 18, male No. 9; Nc, negative control.

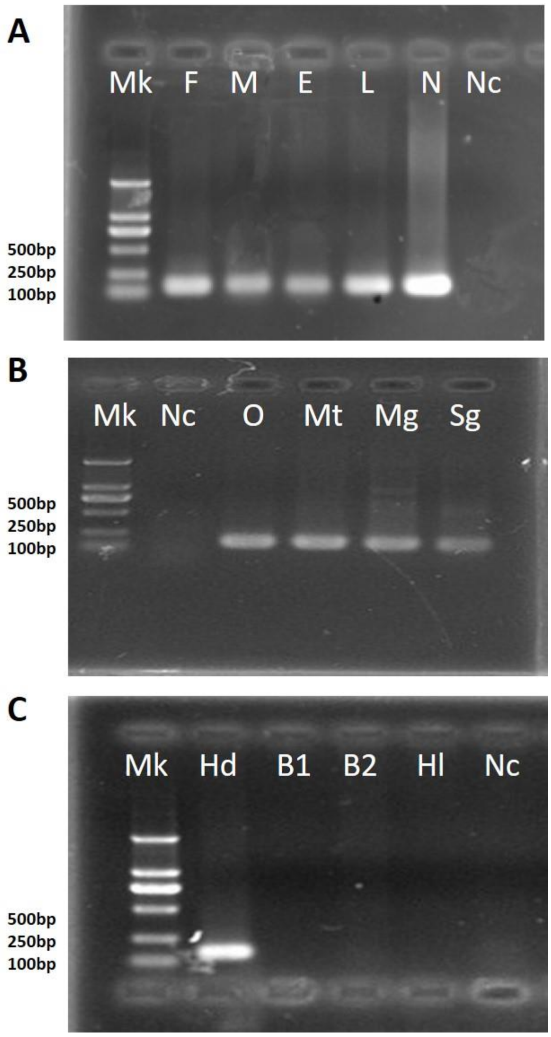

Supplementary Figure 3. The full-length gel of vertical transmission detection of FLEs-Hd at different developmental stages of *H. doenitzi*. Lanes 1 to 7 (A): Mk, DNA Marker; F, females; M, males; E, eggs; L, Larvae; N, nymphs; Nc, negative control. The full-length gel of infection sites detection of FLEs-Hd in different tissues of *H. doenitzi*. Lanes 1 to 6 (B): Mk, DNA Marker; Nc, negative control; O, ovaries; Mt, malpighian tubules; Mg, midguts; Sg, salivary glands. The full-length gel of horizontal transmission detection of FLEs-Hd in host animal and *H. longicornis* collected from the same region as *H. doenitzi*. Lanes 1 to 6 (C): Mk, DNA Marker; Hd, *H. doenitzi*; B1, blood of host animal before tick ingested; B2, blood of host animal after tick ingested; Hl, *H. longicornis*; Nc, negative control.

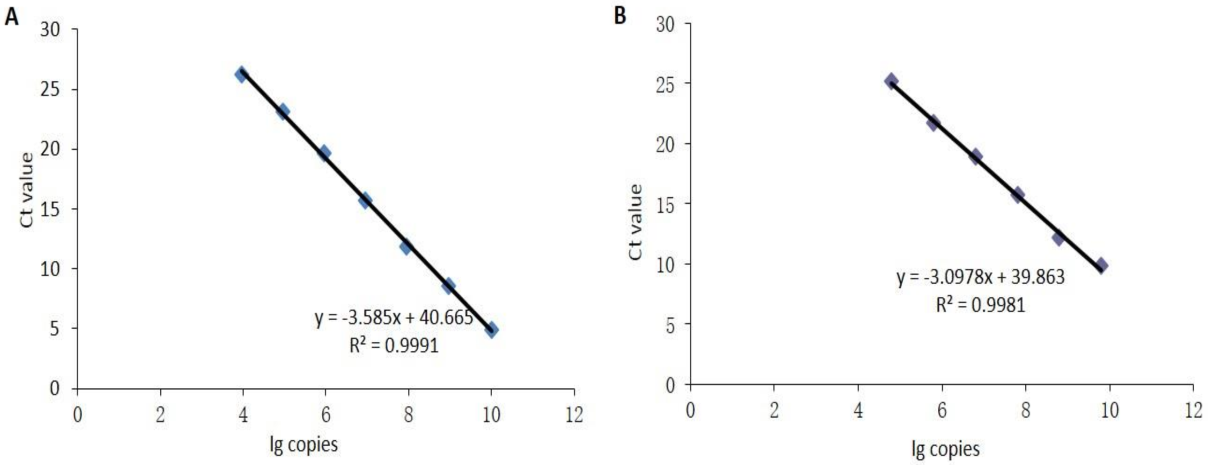

Supplementary Figure 4. The standard curves of actin gene of *H. doenitzi* (A) and 16S rRNA gene of FLEs-Hd (B).
